# The Effect of an Environmentally Relevant Phthalate Mixture on Primordial Germ Cells of *Xenopus laevis* Embryos

**DOI:** 10.17912/micropub.biology.000080

**Published:** 2018-12-03

**Authors:** Nathalie Lancy Momplaisir, Aurora Turgeon, Jodi Flaws, Jing Yang

**Affiliations:** 1 Tuskegee University, Tuskegee, AL, 36088; 2 University of Illinois at Urbana-Champaign, Urbana-Champaign, IL, 61802

**Figure 1:**
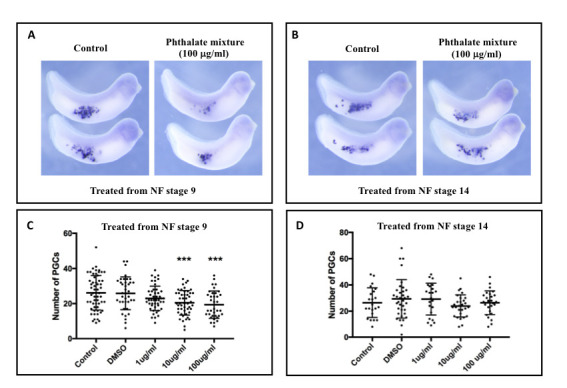
**A** and **B**. Representative images of *pgat* *in situ* hybridization showing *pgat*-positive PGCs (blue dots) in control and phthalate mixture-treated *Xenopus* embryos at NF stage 33 & 34. Embryos were treated with phthalate mixture (100 mg/ml) from NF stage 9 (**A**) and NF stage 14 (**B**), respectively. **C** and **D**. Quantitation of the primordial germ cells in each treatment group. Primordial germ cells were counted from each embryo in each treatment group. The graph shows the abundance of primordial germ cells in embryos exposed to various doses of phthalate mixture from NF stage 9 (**C**) and NF stage 14 (**D**). In order to assess the significance of the data, the unpaired t-test with Welch’s correction was used. *** p<0.005. These experiments were performed three times with similar results.

## Description

Phthalates, being considered endocrine disrupting chemicals, are estrogen-like and anti-androgenic compounds that interfere with hormones which regulate numerous biological processes, including the maintenance of homeostasis and the regulation of development (Kavlock *et al.*, 1996). Toxicological studies have consistently shown that phthalate metabolites are reproductive and developmental toxicants (Jeng, 2014). Some of the significant complications include fertility issues, decreased anogenital distance, low sperm concentration and motility. While the adverse effects of phthalates on the reproductive system have been well-documented, it is currently unclear if exposure to phthalates affects early stage of germline development.

*Xenopus* *laevis* is a widely used vertebrate model system. *Xenopus* embryos develop quickly and externally, offering numerous advantages for toxicological studies. Previous studies have demonstrated that exposure to specific phthalates, such as DEP and DnPP induces malformations, including edema, abnormal gut, abnormal gut coiling (Gardner *et al.*, 2016). It remains unclear if exposure to phthalates affects the development of primordial germ cells (PGCs), the only type of cells in the embryo capable of giving rise to sperms and oocytes in adult animals.

In this study, the effects of an environmentally relevant phthalate mixture on PGCs of the *X. laevis* embryos were assessed. The phthalate mixture was formulated according to the exposure levels found in urine metabolites of pregnant women in the Illinois area (Zhou and Flaws, 2017). This phthalate mixture is composed of 21% di(2-ethylhexyl) phthalate (DEHP), 35% diethyl phthalate (DEP), 15% dibutyl phthalate (DBP), 8% di-isobutyl phthalate (DiBP), 5% benzyl butyl phthalate (BzBP), and 15% di-isononyl phthalate (DiNP). In our study, we treated embryos with various doses of phthalate mixture (1 µg/mL, 10 µg/mL and 100 µg/mL) for 24 hours in two slightly different windows. The first group of embryos were treated with phthalate mixture from Nieuwkoop & Faber (NF) stage 9. At this stage, the genome of developing PGCs remains inactive (Venkatarama *et al.*, 2010). The second group of embryos were exposed to phthalate mixture from NF stage 14, when the genome becomes activated in PGCs (Venkatarama *et al.*, 2010). At NF stage 33 & 34, untreated, vehicle control (DMSO), and phthalate mixture treated embryos were harvested and subjected to whole mount in situ hybridization (WISH) for *primordial germ cell-associated transcript protein* (*pgat*, also known as *Xpat*), a marker for PGCs (Figure 1A and B). After WISH, embryos were cleared in BABB. The number of PGCs in each embryo was counted and statistically analyzed.

**Results**

This experiment demonstrates that exposure to phthalate mixture from NF stage 9 reduced the number of PGCs in a dose-dependent manner (Figure 1C). While the reduction in the 1 µg/mL treatment group was not statistically significant, higher doses of phthalate mixture caused a statistically significant decrease in the number of PGCs (10 µg/ml p=0.0008, and 100 µg/ml p=0.0004). Interestingly, embryos exposed to phthalate mixture from NF stage 14 did not show any reduction in the number of PGCs (Figure 1D). These observations thus suggest that *Xenopus* PGCs are more sensitive to toxicants such as phthalates during earlier embryonic stages, when the development is controlled by maternal factors. Further work is needed to gain a better understanding of the link between zygotic genome activation and the window of sensitivity to the phthalate mixture during PGC development. It will be of interest to determine if phthalate mixture interferes with the function of Nanos1 or Grip2.1, which regulates the number of PGCs during *Xenopus* embryonic development (Lai et al., 2012; Tarbashevich *et al.*, 2007).

## Reagents

*Xenopus* were purchased from Nasco (www.enasco.com). All *Xenopus* procedures were approved by University of Illinois at Urbana-Champaign Institutional Animal Care and Use Committee (IACUC), under animal protocol #17199 and performed in accordance with the recommendations of the Guide for the Care and Use of Laboratory Animals of the National Institutes of Health. *Xenopus* *laevis* embryos were obtained and cultured as described (Sive *et al.*, 2000). All phthalates were obtained from Sigma-Aldrich (www.sigmaaldrich.com). These include di(2-ethylhexyl) phthalate (DEHP, Cat. No. 36735), diethyl phthalate (DEP, Cat. No. 53008), dibutyl phthalate (DBP, Cat. No. 36736), di-isobutyl phthalate (DiBP, Cat. No. 152641), benzyl butyl phthalate (BzBP, Cat. No. 36927), and di-isononyl phthalate (DiNP, Cat. No. 37663). Plasmid for labeling *pgat* *in situ* probe was described (Hudson and Woodland, 1998). Whole amount *in situ* hybridization was performed as described (Rorick *et al.*, 2007).
